# Smooth muscle cell (SMC)-Specific SNRK deletion in mouse causes congenital short bowel syndrome and premature death

**DOI:** 10.1016/j.bbrep.2025.102298

**Published:** 2025-10-13

**Authors:** Chang-Jiang Yu, Liu Ouyang, Junqing An, Ye Ding, Zhi-Xue Liu, Zhi-Ren Zhang, Ming-Hui Zou

**Affiliations:** a Departments of Cardiology and Pharmacy, Harbin Medical University Cancer Hospital, Institute of Metabolic Disease, Heilongjiang Academy of Medical Science, Heilongjiang key laboratory for Metabolic Disorder and Cancer Related Cardiovascular Diseases, Harbin, China; b Center for Molecular and Translational Medicine, Georgia State University, Atlanta, USA; c Departments of Cardiology and Critical Care Medicine, The First Affiliated Hospital of Harbin Medical University, NHC Key Laboratory of Cell Transplantation, Key Laboratories of Education Ministry for Myocardial Ischemia Mechanism and Treatment, Harbin, China; d State Key Laboratory of Frigid Zone Cardiovascular Diseases (SKLFZCD), Harbin Medical University, Harbin, China; e Department of Endocrinology and Metabolism, Tianjin Medical University General Hospital, Tianjin, China

**Keywords:** Congenital short bowel syndrome, SNRK, Circular smooth muscle, Cell proliferation, Intestinal development

## Abstract

Congenital short bowel syndrome (CSBS), a rare gastrointestinal disorder characterized by reduced small intestine length, remains etiologically undefined. This study investigates the role of sucrose nonfermenting 1-related kinase (SNRK) in intestinal smooth muscle cells (SMCs) during development and in adult homeostasis using two mouse models: a congenital SMC-specific Snrk knockout (SNRK-SMKO) and an inducible adult knockout. SNRK-SMKO mice died prematurely and exhibited shortened intestines, dilated lumens, and a thinner circular muscle layer without evidence of increased apoptosis. In contrast, adult-onset Snrk deletion had no apparent effect. Western blot analysis revealed elevated SMC contractile proteins in neonatal (postnatal day 3.5) SNRK-SMKO colons, which declined during maturation. 5-Ethynyl-2′-deoxyuridine (EdU) incorporation assays showed persistently reduced SMC proliferation in the circular muscle layer of both small intestine and colon across postnatal developmental stages. These results establish SNRK as a critical regulator of intestinal SMC proliferation during development, with its deficiency underlies CSBS-like pathology.

## Highlights

• Congenital smooth muscle cell (SMC)-specific Snrk knockout in mice induces neonatal short bowel syndrome.
• Adult-onset Snrk deletion has no apparent effect.
• Snrk deficiency impairs intestinal SMC proliferation and alters contractile-protein expression.

## 1 Introduction

Congenital short bowel syndrome (CSBS) is a rare gastrointestinal disorder, first described by Hamilton et al. [1] in 1969 CSBS patients were born with a significantly shortened small intestine, often presenting with vomiting, chronic diarrhea, and failure to thrive [2,3]. CSBS is primarily reported in the form of case reports. Incorporating the 61 cases summarized in a review paper by Elisa Negri et al. [3], we found that only 80 patients were recorded from 1969 to the present. Due to advancements in nutrition and intestinal rehabilitation, the survival rate of CSBS patients increased from 28.5 % before 2008 to 75 % in the period following 2008 [3,4]. However, the exact etiology of CSBS remains elusive. The familial occurrence provides evidence of the genetic basis in CSBS patients. Following the work of Van der Werf et al. [5], several autosomal recessive mutations in coxsackie and adenovirus receptor-like membrane protein (CLMP) were discovered in patients with CSBS [6,7]. Loss-of-function of Clmp by morpholino injection in zebrafish resulted in significant shortening of the intestine and developmental delay, similar to the clinic phenotype of CSBS [5]. A mouse model with a global knockout of Clmp also exhibited intestinal malrotation and early lethality, despite having a normal intestinal length [8]. Filamin A (FLNA) mutation is another genetic defect involved in the development of CSBS, which is related to the X-linked recessive inheritance pattern [[9], [10], [11]]. While these findings brought new insights into the pathogenesis of CSBS, the underlying mechanisms by which CLMP and FLNA contribute to intestinal elongation are still unknown. On the other hand, the potential mechanisms for the development of CSBS in patients without identified mutations in these genes are completely unknown.

Sucrose nonfermenting 1-related kinase (SNRK) is serine/threonine kinase and a member of AMP-activated protein kinase (AMPK) subfamily. It contains a conserved T-loop threonine residue (Thr173) at the *N*-terminal which can be phosphorylated by liver kinase B1 (LKB1) to induce activation [12]. SNRK was first identified from adipocyte-like cells that had differentiated from rat 3T3-L1 fibroblast [13], but it exhibited widespread expression across various tissues of human, rat, and mouse [13,14]. Subsequent studies confirmed that SNRK was involved in both developmental processes and various diseases. SNRK played a crucial role in the early development of angioblast and artery-vein specification in zebrafish model [15,16]. In a hindlimb ischemia mouse model, Lu et al. [17] reported that endothelial SNRK promoted angiogenesis by activating β1 integrin (ITGB1)-mediated endothelial cell migration. SNRK was shown to act as an inflammation suppressor and was implicated in adipose inflammation [[18], [19], [20]]. As well as inflammation induced by angiotensin II in the kidney and heart [21,22]. SNRK expression was found to be decreased in human colon cancer tissues, while overexpression of SNRK inhibited colon cancer cell proliferation by upregulating calcyclin-binding protein (CacyBP) and promoting β-catenin degradation [23]. In ovarian cancer, reduced SNRK expression was corelated with advanced-stage disease and metastasis [24]. Of note, emerging evidence indicates that SNRK plays a critical role in heart development and function, primarily involved in regulating mitochondrial homeostasis [[25], [26], [27]], inflammation [22], and the DNA damage response [28]. However, there are no reports or documentation regarding the function of SNRK in smooth muscle physiology and pathology.

In this study, we use both congenital and adult-onset SMC-specific Cre drivers to explore the role of SNRK in both developing and adult mice. The congenital deletion of Snrk gene in SMC caused lethality among mice shortly after birth, concomitant with noticeable shortening and distension across the entire intestinal tract. Phenotypic studies showed that Snrk deficiency leads to reduced SMC proliferation and a thinner circular smooth muscle layer in the intestinal tract. In contrast, inducible ablation of Snrk in SMC in adult mice exhibited regular intestinal morphology and function. Collectively, these findings offer compelling evidence for a previously unrecognized role of SMC Snrk in regulating intestinal development.

## 2 Methods

### 2.1 Animal

All animal procedures were approved by the Animal Care and Use Committee of Georgia State University (Protocol A21053) were conducted in accordance with the Guide for the Care and Use of Laboratory Animals published by the National Institutes of Health.

Snrk^flox/flox^ mice were kindly provided by Dr. Ramani Ramchandran at Medical College of Wisconsin [25]. Smooth muscle cell (SMC)-specific heterozygous Snrk gene deletion (Snrk^flox/wt^-Myh11-Cre) mice were generated by crossbreeding Snrk^flox/flox^ mice with smooth muscle myosin heavy chain 11 Cre transgenic mice (Myh11-Cre/EGFP, 007742, Jackson Laboratory, ME, UA). Then, female Snrk^flox/flox^ mice were mated with male Snrk^flox/wt^-Myh11-Cre mice to generate SMC-specific homozygous Snrk gene deletion (Snrk^flox/flox^-Myh11-Cre) mice. Both male and female Snrk^flox/flox^-Myh11-Cre mice were used in this study. Identification of a vaginal plug was regarded as day 0.5 of gestation. Inducible SMC-specific Snrk gene deletion mice were achieved by pairing female Snrk^flox/flox^ mice with male Myh11-CreER^T2^ mice (019,079, Jackson Laboratory). 6- to 8-week-old male Snrk^flox/flox^-Myh11-CreER^T2^ mice were injected intraperitoneally with 20 mg/kg/d Tamoxifen (T5648, Sigma-Aldrich) for 5 consecutive days to induce Cre-mediated Snrk gene deletion in SMC. All genetic models had the C57BL/6 background. The animals were housed in the animal facility at Georgia State University with a controlled environment on a 12-h light/dark cycle. Mice had free access to sterilized food and water.

### 2.2 Anatomical measurements

Body and organ weights were recorded when the mice were euthanized at the indicated time points. The entire gastrointestinal tract (from esophagus to anus) was isolated and taken image. The length of small intestine and colon was measured. The width of the intestinal tract was expressed as a mean of 4 measurements from the indicated segments.

### 2.3 Assessment of small intestine transit

Small intestine transit assay was performed as described previously with minor modification [29]. The mice were fasted for 4–6 h, then 100 μL of the non-absorbable Evans Blue dye (5 % suspended in 0.9 % NaCl solution with 0.5 % methyl cellulose) was applied orally at single dose. 15 min after gavage, the mice were euthanized and the whole intestine tract was immediately removed and completed the image taking. Length of the small intestine and distance traveled by the dye were measured. Results are expressed as a percentage of the length covered by the dye over the total small intestinal length.

### 2.4 Histological analysis and immunofluorescence

Small intestines and colons were isolated from mice and lumen feces were removed. The duodenum, jejunum, ileum, and colon sections were identified according to previously published paper [30]. 0.5–1 cm length of tissue from each region was prepared and immediately fixed in 10 % buffered formalin (SF100-4, Fisher Scientific, PA, USA) at 4 °C for 24 h. In certain samples, the small intestine and colon were split open under a dissection microscope using microsurgical scissors and tweezers. The outer wall of the open intestinal tract was attached to a strip of filter paper and incubated in the fixation solution. After fixation step, the intestinal swiss roll was prepared. Tissues were embedded in paraffin or optimal cutting temperature (OCT) compound. Paraffin sections (4 μm) of intestinal tissues were stained with H&E. The thickness of smooth muscle layer of each mouse was calculated as a mean value from 12 to 18 separate positions in each section. Cryosections (8 μm) of intestinal tissues were stained with Phalloidin (#13054, CST) to label F-actin. For immunofluorescence studies, paraffin-embedded slides were subjected to de-waxing, rehydrating, and heat antigen retrieval in sodium citrate buffer. Then the slides were permeabilized with 0.2 % Triton X-100, and blocked with protein block goat serum (BioGenex, CA). Finally, the slides were incubated with antibodies against α smooth muscle actin (αSMA), myosin light chain kinase (MLCK), Calponin 1, or Cleaved Caspase-3 at 4 °C overnight and then exposed to fluorescence conjugated secondary antibodies (Alexa Fluor®, Invitrogen). Nuclei were stained with DAPI (D9564, Sigma-Aldrich). Images were captured using a microscope (IX73, OLYMPUS) and quantified using Image J software (version 1.54).

### 2.5 Western blot

Western blot analysis was performed as preciously described [31]. The smooth muscle layer tissues from mice stomach, colon, and bladder were isolated under dissection microscope with microsurgical scissors and tweezers. But whole colon tissues from mice aged postnatal 3.5 d and 7.5 d were collected to prepare tissue lysate. Proteins were separated by SDS-PAGE and transferred to nitrocellulose membranes (Millipore, MA). Proteins detected by antibodies were visualized using an enhanced chemiluminescence detection system (GE Healthcare, WI) and quantified using image J software. Antibodies used in this study are shown as follows. Anti-SNRK (NBP1-83668, Novus Biologicals), anti-voltage-dependent L-type calcium channel subunit α 1C (CACNA1C, ab84814, Abcam), *anti*-MLCK (M7905, Sigma-Aldrich), *anti*-Myocardin (MAB4028, Novus Biologicals), *anti*-αSMA (ab7817, Abcam), Calponin 1 (17,819, Cell Signaling Technology), anti-smooth muscle protein 22α (SM22α, 40,471, Cell Signaling Technology), *anti*-Cyclin D1 (55,506, Cell Signaling Technology), anti-proliferating cell nuclear antigen (PCNA, 10205-2-AP, Proteintech), anti-Cleaved Caspase-3 (9661, Cell Signaling Technology), *anti*-β-Actin (sc-47778, Santa Cruz).

### 2.6 Proliferation and apoptosis assays

Click-iT™ Plus EdU Cell Proliferation Kit (C106377 and C106339, Thermo Fisher Scientific) was used to labeled newly synthesized DNA. 10 mM EdU in PBS was injected intraperitoneally to mice aged postnatal 6.5 d or 13.5 d or pregnant female mice (17.5 d post coitum) at a single dose of 100 μL per 10 g of body weight for 24 h. For mice aged postnatal 3.5 d, EdU was injected subcutaneously for 4 h. Then, the intestine tissues were collected, and tissue sections were prepared. EdU incorporation assay was performed according to the manufacturer's instructions and previously published paper [32]. EdU-positive cell number in each mouse was calculated as a mean value from 1 to 3 fields in each section. The sections were also stained with DAPI to label nuclei and αSMA to show the smooth muscle. Cell apoptosis was performed by using Roche TUNEL assay kit (11767305001 and 11767291910, TUNEL enzyme and TUNEL label mix).

### 2.7 Statistical analysis

All data were presented as mean ± SEM. Statistical analyses were performed using GraphPad Prism version 9.5.1 (CA, USA). The Shapiro-Wilk test was used to assess data normality. Survival analysis was conducted using the log-rank test. Statistical significance was determined using either a two-tailed unpaired Student's *t*-test for parametric data or the Mann-Whitney *U* test for non-parametric data. A *P*-value of <0.05 was considered statistically significant.

## 3 Results

### 3.1 Congenital deletion of snrk in smooth muscle cell results in shortened intestinal tract and lethality

**Fig. 1:**
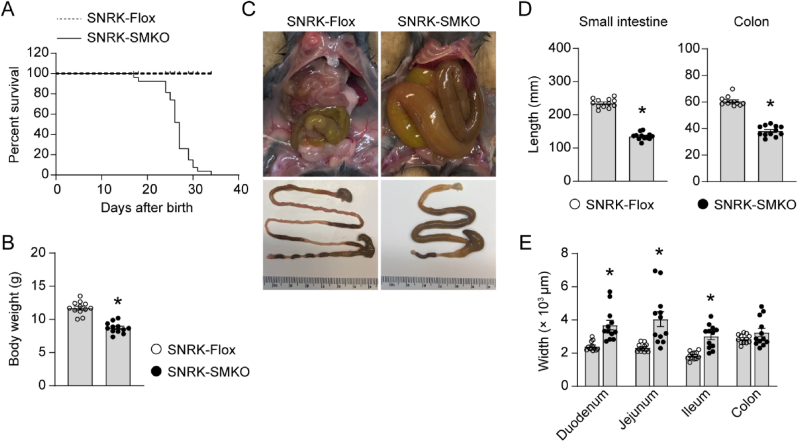
Smooth muscle cell-specific Snrk gene deletion results in shortened intestinal tract and lethality. (A) Percent survival of SNRK-Flox and SNRK-SMKO mice after birth. 27 mice were recorded for each group. **(B)** Body weights of SNRK-Flox and SNRK-SMKO mice under 25 d of age. **(C)** Macroscopic images of the opened abdominal cavity (top) and dissected entire gastrointestinal tract (bottom). **(D)** The lengths of small intestine (from pylorus to ileocecal junction) and colon (from cecum to anus). **(E)** The widths of duodenum, jejunum, ileum, and proximal colon. N = 12 mice/group. ∗*P* < 0.05 *vs*. SNRK-Flox group.

To explore the role of Snrk in smooth muscle physiology, we generate smooth muscle cell (SMC)-specific Snrk gene deletion mice by introduction of Myh11-Cre in Snrk^flox/flox^ (hereafter referred as SNRK-Flox) mice. However, homozygous Snrk gene knockout (Snrk^flox/flox^-Myh11-Cre; hereafter referred as SNRK-SMKO) mice died in a short period after birth (Fig. 1A). The median survival time was 27 d. There is no sex bias for this phenotype. To better characterize the phenotype shown in SMKO mice, mice were collected for measurement at 25 d after birth, or even earlier if they met the humane endpoints. Compared with SNRK-Flox mice, SNRK-SMKO mice showed significantly lower body weight and organ weights (Fig. 1B and Fig. S1) and suffered from diarrhea, dehydration, and other pathological syndromes. As shown in Fig. 1C–E, macroscopic examination revealed a severe dilation in part or the entire intestinal tract. The length of small intestine and colon in SNRK-SMKO mice was significantly shorter than that of SNRK-Flox mice. There is no significant difference in colon width observed in the statistical results due to large variance. However, the dilated colon in SNRK-SMKO mice was always accompanied by accumulated fecal matter. In contrast, SNRK-Flox mice showed normal and healthy colon with well-formed fecal pellets.

**Fig. 2:**
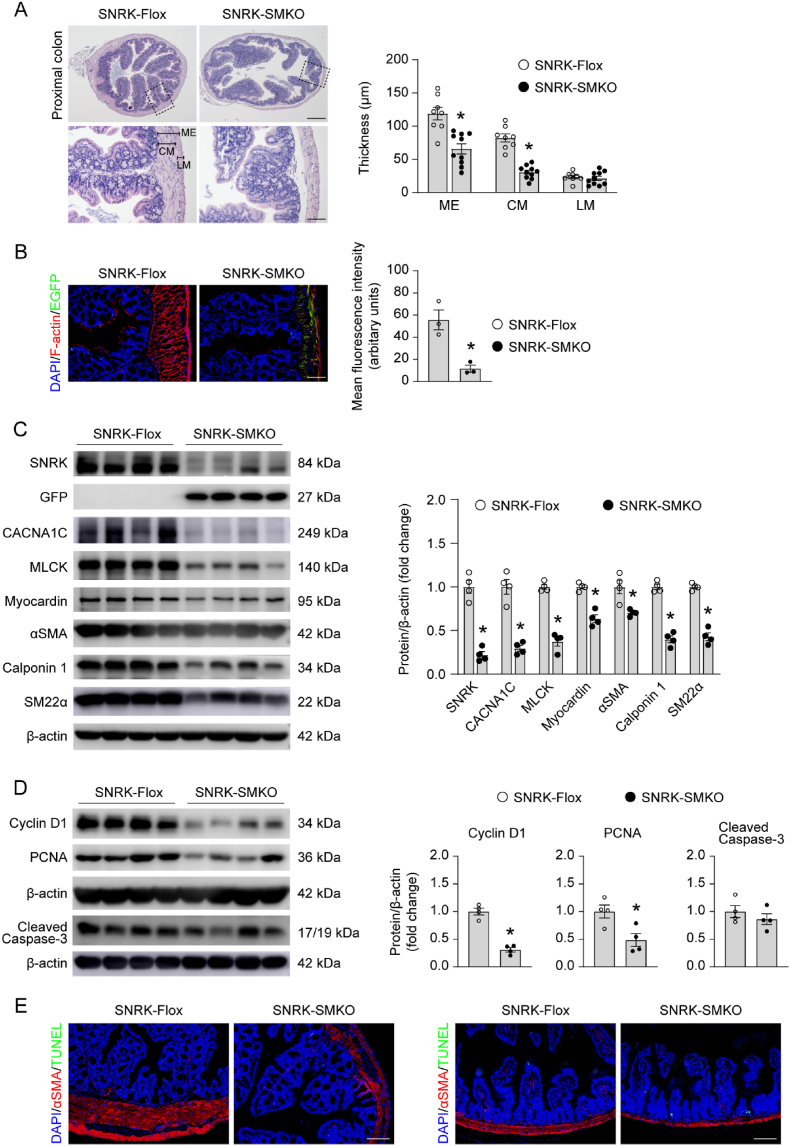
Smooth muscle cell-specific Snrk gene deletion decreases the thickness of circular smooth muscle layer and the expression of contractile markers. (A) Left: Representative images of H&E staining of proximal colonic cross sections from SNRK-Flox and SNRK-SMKO mice under 25 d of age. Scale bar of top images, 500 μm. Scale bar of bottom images, 100 μm. Right: Quantified thickness of muscularis externa (ME), circular muscle layer (CM), and longitudinal muscle layer (LM). N = 8–10 mice/group. **(B)** Representative images and quantification of F-actin staining (red) in the cryosections of proximal colon of SNRK-Flox and SNRK-SMKO mice. Spontaneous green color indicates Myh11-Cre/EGFP in SMC of SNRK-SMKO mice. DAPI staining for nuclei (blue). Scale bars, 100 μm. N = 3 mice/group. **(C)** Representative western blots and quantification of the proteins expression involved in contractility in colon smooth muscle isolated from SNRK-Flox and SNRK-SMKO mice. N = 4 mice/group. **(D)** Western blot analysis for the expression of Cyclin D1, PCNA, and Cleaved Caspase-3 in colon smooth muscle isolated from SNRK-Flox and SNRK-SMKO mice. N = 4 mice/group. **(E)** Representative images of TUNEL staining (green), immunofluorescence staining of αSMA (red), and DAPI staining for nuclei (blue) in the proximal colon and jejunum from SNRK-Flox and SNRK-SMKO mice. ∗*P* < 0.05 *vs*. SNRK-Flox group.

As such, we focused our analysis on the intestinal tract of mice. Histological sections at different regions of the intestinal tract revealed an obvious remodeling in the intestinal wall. The thickness of muscularis externa and circular smooth muscle layer was thinner in the proximal colon, as well as the small intestine in SNRK-SMKO mice (Fig. 2A and Fig. S2A–C). The thickness of longitudinal smooth muscle layer showed no significant changes. F-actin was labeled with fluorescent phalloidin. A reduced F-actin signal was detected in the smooth muscle layer of proximal colon in SNRK-SMKO mice (Fig. 2B), potentially impacting intestinal contractile properties. Then, we assessed the expression levels of several proteins involved in regulating SMC contraction. Western blots results showed all the contractile markers, including CACNA1C, MLCK, Myocardin, αSMA, Calponin 1, and SM22α were down-regulated in the colon smooth muscle of SNRK-SMKO mice, compared to those in SNRK-Flox mice (Fig. 2C). The expression levels of these markers also decreased to some extent in the bladder, a distensible hollow muscular organ (Fig. S3A). The expression levels of the proliferating markers Cyclin D1 and PCNA were decreased in the colon smooth muscle of SNRK-SMKO mice. However, active caspase-3 was not changed in SNRK-SMKO mice (Fig. 2D). In addition, although a few cells with TUNEL-positive signals were observed in the small intestine mucosa of SNRK-SMKO mice, such signals were undetectable in the smooth muscle layer across all groups (Fig. 2E). The expression of Cyclin D1 was also decreased in the bladder of SNRK-SMKO mice, while the expression of active caspase-3 was not significantly changed (Fig. S3B). Taken together, these data suggest that Snrk may play a crucial role in intestinal development and is essential for mouse growth and survival.

### 3.2 Conditional deletion of snrk in smooth muscle cell of adult mice shows normal intestinal morphology and function

**Fig. 3:**
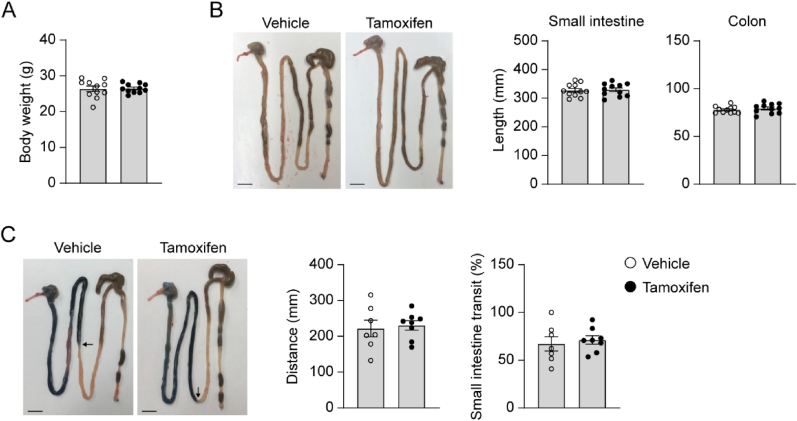
Inducible smooth muscle cell-specific deletion of SNRK in adult mice display normal intestinal morphology and motility. (A) Body weights were measured in vehicle- or tamoxifen-treated Snrk- Myh11-CreER^T2^ mice at 16 weeks of age. N = 11 mice/group. **(B)** Macroscopic images of the dissected entire gastrointestinal tract and the quantification of the length of small intestine and colon. Scale bars, 1 cm. N = 11 mice/group. **(C)** Representative images showing small intestinal transit measured by recording the migrating distance of Evans blue (black arrows) and calculated percentage of the length covered by Evans blue over the total length of small intestine 15 min after application. Scale bars, 1 cm. N = 7–8 mice/group.

Since congenital SNRK-SMKO mice did not survive for long periods, inducible SMC-specific Snrk gene deletion mouse line was generated to further explore whether Snrk in SMC plays important roles in adult mice. We crossed female Snrk^flox/flox^ mice with male mice that express tamoxifen-inducible Cre recombinase under the control of Myh11 promoter (Myh11-CreER^T2^). Deficiency of Snrk protein expression in smooth muscle of mouse colon was confirmed by Western blot (Fig. S4A). In contrast to congenital Snrk gene deletion (SNRK-SMKO), there is no overt intestinal phenotype in inducible Snrk gene knockout mice. Tamoxifen-treated group showed similar gross parameters with vehicle-treated control group, as indicated by measurement of body weight, intestinal morphological and histological characteristics, as well as SMC contraction-associated proteins expression (Fig. 3A and B, Fig. S4B and C). Moreover, no change occurred in tamoxifen-treated mice when evaluating small intestine transit (Fig. 3C), suggesting normal intestinal motility. Taken together, the loss of Snrk in SMC of adult mice shows no pathological phenotype.

### 3.3 Smooth muscle cell-specific snrk gene deletion induces intestinal developmental disorders

**Fig. 4:**
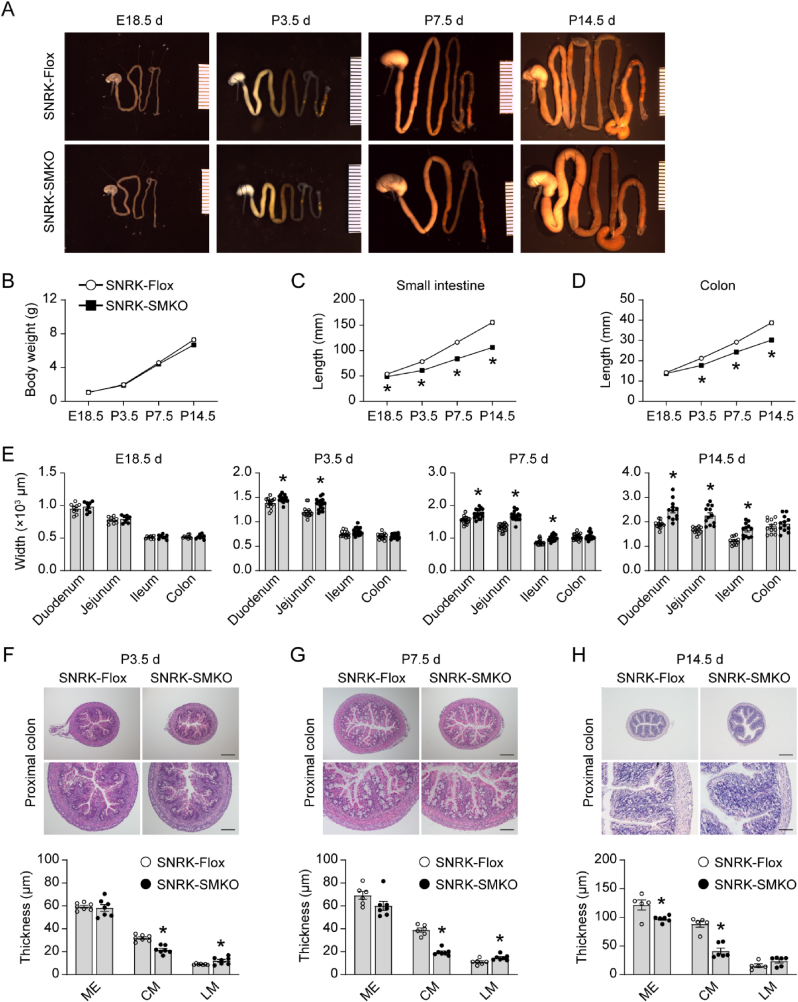
Characterization of smooth muscle cell-specific SNRK gene deletion on intestinal development. (A) Morphological changes of the gastrointestinal tract between SNRK-Flox and SNRK-SMKO mice at ages from embryonic 18.5 d (E18.5 d) to postnatal 14.5 d (P14.5 d). **(B)** Body weights of SNRK-Flox and SNRK-SMKO mice at the indicated time points. **(C and D)** The lengths of small intestine **(C)** and colon **(D)**. **(E)** The widths of the intestinal tracts, including duodenum, jejunum, ileum, and colon of SNRK-Flox and SNRK-SMKO mice at the indicated time points. N = 8–9 mice/group in mice aged E18.5 d N = 14–16 mice for group P3.5 d N = 18–20 mice/group in mice aged P7.5 d N = 12 mice/group in mice aged P14.5 d. **(F–H)** Representative images of H&E-stained proximal colon in the indicated groups (top), and the quantification of the thickness of muscularis externa (ME), circular muscle layer (CM), and longitudinal muscle layer (LM) (bottom). N = 7 mice for group P3.5 d N = 6–7 mice/group in mice aged P7.5 d N = 5–6 mice/group in mice aged P14.5 d.

Based on the findings mentioned above, we speculate that Snrk plays an important role in intestinal development. To better understand the role of Snrk during intestinal development, we examined the effects of Snrk deletion at different stages of mouse growth (Fig. 4A). Body weights (Fig. 4B) and organ weights (Fig. S5A and B) showed no significant changes between SNRK-Flox and SNRK-SMKO mice at embryonic (E) 18.5 d, postnatal (P) 3.5 d, 7.5 d, and 14.5 d. However, SNRK-SMKO mice exhibited a shorter small intestine as early as E18.5 d, with no change on colon length (Fig. 4A–C and D). Although the length of the small intestine and colon increased as mice grew, SNRK-SMKO mice consistently exhibited shorter lengths compared to SNRK-Flox mice (Fig. 4A–C and D). Meanwhile, there was also a progressive dilation observed in the small intestine of SNRK-SMKO mice, extending from the duodenum to the ileum (Fig. 4E). Individual values of body weight and intestinal length were shown in Fig. S5C–F. Histological H&E staining revealed that the loss of Snrk in the SMC decreased the thickness of muscularis externa in the proximal colon as mice grew, showing significant change at 14.5 d after birth (Fig. 4F–H). Compared to age-matched SNRK-Flox mice, the circular muscle layer was consistently thinner in SNRK-SMKO mice, while the longitudinal muscle layer was relatively thicker. Decreased thickness of circular muscle layers was also observed in the small intestine of SNRK-SMKO mice (Fig. S6A–C).

**Fig. 5:**
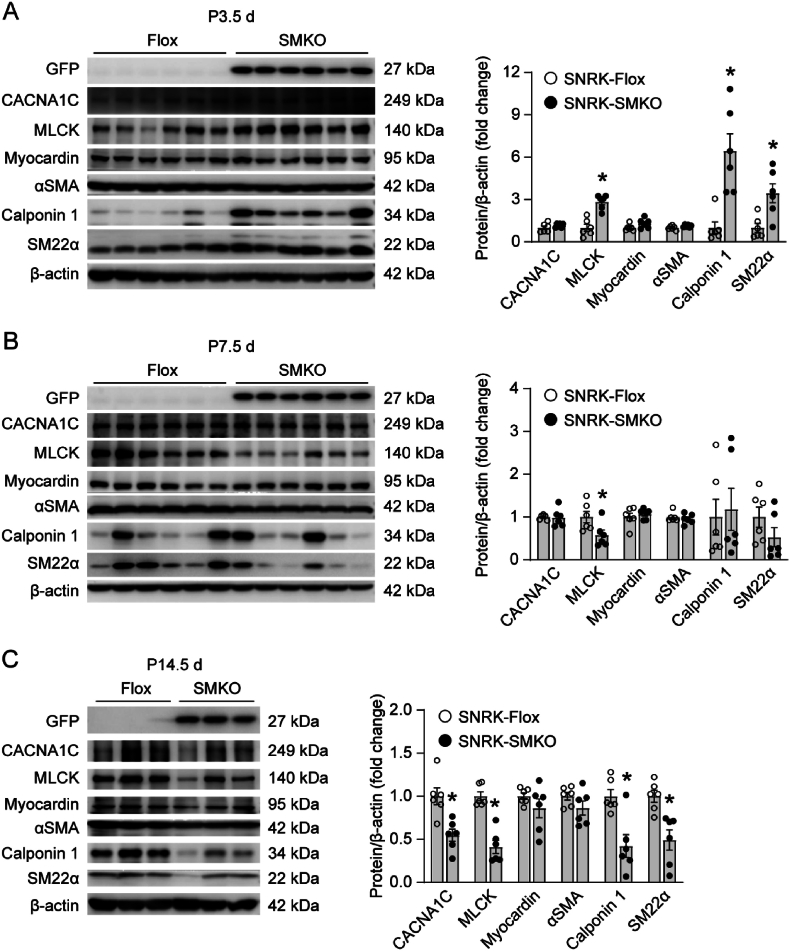
Smooth muscle cell-specific Snrk gene deletion results in dynamic changes of the expression of smooth muscle contractile markers in the colon. **(A**–**C)** Representative western blots and quantification of the expression levels of smooth muscle contractile makers in the colon tissues of SNRK-Flox and SNRK-SMKO mice at ages of P3.5 d **(A)** and P7.5 d **(B)**, or in the smooth muscle isolated from colon of mice aged P14.5 d **(C)**. N = 6 mice/group. ∗*P* < 0.05 *vs*. SNRK-Flox group.

We next investigated the protein expression levels of SMC contractile markers in the colon (Fig. 5A–C). The expression levels of CACNA1C, Myocardin, and αSMA were comparable between SNRK-Flox and SNRK-SMKO mice at P3.5 d (Fig. 5A). However, the expression levels of MLCK, Calponin 1, and SM22α were significantly increased in SNRK-SMKO mice compared to SNRK-Flox mice. Interestingly, only a significant decreased MLCK expression was observed in the colon of SNRK-SMKO mice at P7.5 d (Fig. 5B). Then, most of these contractile markers, including CACNA1C, MLCK, Calponin 1, and SM22α were decreased in SNRK-SMKO mice at P14.5 d (Fig. 5C). These results suggest that loss of Snrk in SMC leads to dynamic changes of the expression of contractile proteins in the colon of mice during the growth and development process. Although the proteins expression profiles were not entirely consistent with that in the colon, similar changes were observed in the stomach (Fig. S7A–C) and bladder (Fig. S8A–C) of SNRK-SMKO mice compared with SNRK-Flox mice.

### 3.4 Smooth muscle cell-specific SNRK gene deletion inhibits circular SMC proliferation

**Fig. 6:**
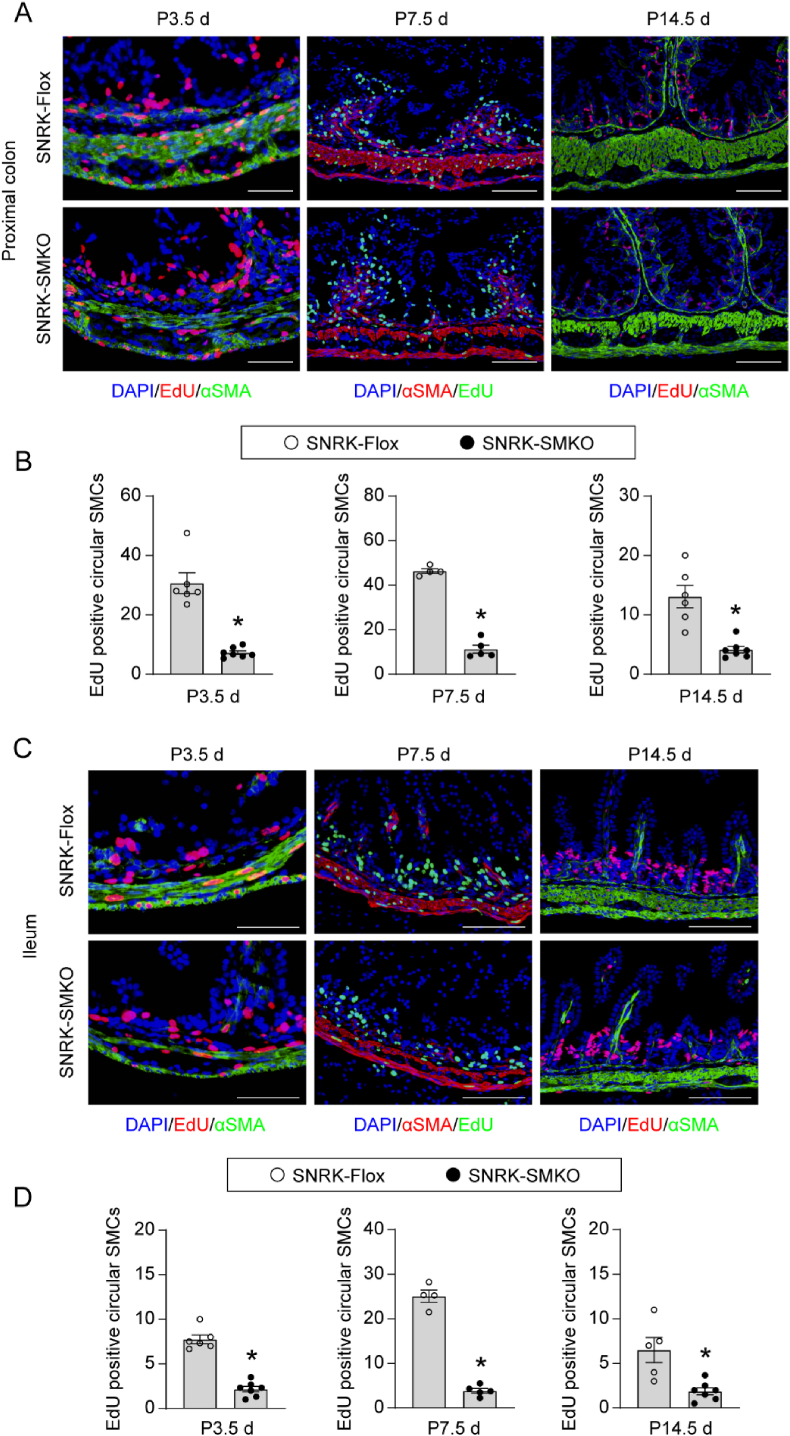
Smooth muscle cell-specific SNRK gene deletion inhibits circular SMC proliferation. (A) Representative images of EdU staining in the proximal colon of SNRK-Flox and SNRK-SMKO mice at ages of P3.5 d, P7.5 d, and P14.5 d. SMC was stained with αSMA. DAPI was used to label nuclei. **(B)** Quantification of EdU-positive cell numbers in the colon circular muscle layer of each high-power field of the indicated groups. N = 6–7 mice/group in mice aged P3.5 d N = 4–5 mice/group in mice aged P7.5 d N = 6–7 mice/group in mice aged P14.5 d ∗*P* < 0.05 *vs*. SNRK-Flox group. **(C)** Representative images of EdU staining in the ileum of SNRK-Flox and SNRK-SMKO mice at ages of P3.5 d, P7.5 d, and P14.5 d. SMC was stained with αSMA. DAPI was used to label nuclei. **(D)** Quantification of EdU-positive cell numbers in ileum circular muscle layer of each high-power field of the indicated groups. N = 6–7 mice/group in mice aged P3.5 d N = 4–5 mice/group in mice aged P7.5 d N = 5–7 mice/group in mice aged P14.5 d ∗*P* < 0.05 *vs*. SNRK-Flox group.

The loss of SMC in the circular layer of SNRK-SMKO mice prompts us to speculate that Snrk may regulate SMC proliferation. 5-Ethynyl-2′-deoxyuridine (EdU) is a thymidine analog that can be incorporated into replicating DNA for detection of de novo DNA synthesis. It is a sensitive and reliable method to measure cell proliferation [32,33]. A significant decrease in cell proliferation, as indicated by less EdU-positive cell numbers, was detected in colon circular smooth muscle of SNRK-SMKO mice at P3.5 d, P7.5 d, and P14.5 d (Fig. 6A and B). Consistently, the loss of Snrk in SMC also inhibited circular smooth muscle cell proliferation in small intestine at different postnatal stages (Fig. 6C and D). Whereas no apoptosis was found in colon smooth muscle of both SNRK-Flox and SNRK-SMKO mice, as indicated by the expression of active caspase-3 (Fig. S9A and B).

## 4 Discussion

CSBS is a severe and rare gastrointestinal disorder whose etiology remains unknown. To the best of our knowledge, this study represents the initial documentation that SMC-expressed Snrk is essential for intestinal development and survival of mice. We observed early lethality with obviously shortened intestine and severe intestinal distension in mice with congenital SMC-specific Snrk gene deletion. Conversely, conditional deletion of Snrk in SMC of adult mice showed no abnormalities in growth, intestinal morphology, or function. Thorough phenotypic studies revealed that Snrk deficiency induces a reduction in the thickness of circular smooth muscle via the inhibition of SMC proliferation in both the small intestine and colon. Despite the persistent shortened intestine observed in SNRK-SMKO mice, the expression levels of SMC contractile proteins displayed dynamic changes. Our findings underscore the significance of Snrk in intestinal SMC physiology, offering new insight into understanding the development and progression of CSBS.

SNRK is a serine/threonine kinase that exhibits widespread expression in mammalian cells including endothelial cells, SMCs, and cardiomyocytes [14,25]. Global Snrk gene knockout mice displayed enlarged heart in embryos and could not survive beyond 24 h after birth [25]. Additionally, the homozygous loss of Snrk in cardiomyocytes resulted in lethality between 8 and 10 months of age, because of metabolic disorders in the cardiac tissues. These results emphasize an essential role of Snrk during heart development and the maintenance of cardiac metabolic homeostasis. Myh11 is considered as the most specific marker for differentiated SMC [34]. In this study, the Myh11-driven Cre-mediated deletion of Snrk in SMC also led to postnatal lethality in mice. SNRK-SMKO mice displayed progressive developmental defects, characterized by a shortened and dilated intestinal tract, along with a significant loss of body and organ weights. These results unveil an overlooked intestinal developmental abnormality in global Snrk gene knockout mice, closely resembling those observed in human patients with CSBS [3]. Beyond the observed heart developmental abnormality, neonatal mice with a global Snrk knockout also displayed impairments in intestinal lipid absorption [25]. Epithelial and mesenchymal cells play diverse regulatory roles in the organogenesis and maintenance of intestinal homeostasis [[35], [36], [37]]. Yang et al. [38] recently showed that ciliary Hedgehog signaling in intestinal mesenchymal cells patterns the circumferential smooth muscle and promotes intestinal elongation. Given the aforementioned information, it would be intriguing to investigate whether Snrk expression in other cell types within the intestinal tract regulates intestinal development.

A rapid lethal phenotype caused by colonic pseudo-obstruction has been shown in SMC-specific loss of serum response factor (SRF) [39,40] or YAP/TAZ [41], primarily attributed to contractile dysfunction in intestinal SMCs. To further explore the impact of Snrk loss on intestinal contractility in adult mice, an inducible adult-onset SMC-specific Snrk deletion mouse model was generated. These mice are viable with no overt phenotype. Mice with Snrk deficiency specifically in SMC showed comparable intestinal length and motility. Given the striking phenotype in the congenital SNRK-SMKO mice, it is plausible that a postnatal sensitive window exists during which SNRK is required for intestinal growth and maturation. In adults, compensation by other AMPK-related kinases or downstream SNRK effectors may also mitigate for the impact of SNRK loss, preserving contractility and tissue homeostasis under baseline conditions. Notably, Maiko Yamaji et al. [42] reported that conditional SMC-specific Nrp1 deficiency reduced intestinal length by 6 months, with intestinal pseudo-obstruction not manifesting until 18 months. Thus, a phenotype may emerge only over longer time frames or under stress, scenarios not examined in the present study. Looking back on the congenital Snrk knockout mice, the unchanged expression of active caspase-3 and the absence of detectable TUNEL staining in intestinal smooth muscle argue against the involvement of apoptosis mediated by Snrk deficiency. Hence, we directed our study towards examining the consequences of Snrk deficiency throughout intestinal development. The results showed that the length of small intestine and colon displayed significant reduction in SNRK-SMKO mice at embryonic (E) 18.5 d and postnatal (P) 3.5 d, respectively. In comparison with SNRK-Flox mice, the difference gradually enlarges as mice grow. Despite the shortened intestinal length shown in SNRK-SMKO mice, there has no significant changes of both body and organ weights between SNRK-Flox and SNRK-SMKO mice at age from E18.5 d to P14.5 d. These findings indicate that the intestinal shortening phenotype at this period is not reflective of global growth defects. However, quantitative assessment of segmental motility, specifically peristaltic frequency and contractile force, in both the congenital and inducible SNRK-KO models would substantially strengthen the conclusions regarding SNRK's role in gut motility. Because the enteric nervous system (ENS) and interstitial cells of Cajal (ICC) are key regulators of intestinal motility [43,44], the absence of overt ENS abnormalities in the present study does not exclude subtler defects. More sensitive whole-mount analyses of myenteric and submucosal plexuses, together with ICC-focused evaluations, are therefore warranted. We hypothesize that SNRK in smooth muscle may modulate ENS/ICC-mediated control of contractility and overall gut motility, and we will test this directly in future work.

Circular smooth muscle forms as the first smooth muscle layer during gut organogenesis, then spontaneous contractions of this layer align the second longitudinal layer [45]. Several mouse models have been reported with a shortened intestine, often associated with genes linked to the contractility of circular smooth muscle cells [46]. Indeed, the partial ablation of the smooth muscle, the disruption of circumferential residual stress through longitudinal incision of the intestinal wall, and the pharmacological inhibition of smooth muscle contraction all resulted in decreased longitudinal growth of the intestine [38,47]. Consistent with these findings, SNRK-SMKO mice consistently exhibited a thinner circular smooth muscle layer across various developmental stages, paralleled by a reduction in intestinal length. Upon further examination, the protein expression levels of smooth muscle contractile markers exhibited dynamic changes throughout postnatal intestinal development. Unlike the overall decrease in contractile proteins levels observed in the colon smooth muscle of SNRK-SMKO mice at ages P14.5 d and P25.5 d, a significant upregulation in the expression levels of MLCK, Calponin 1, and SM22α was detected in the colon of mice at age P3.5 d. At the age of P7.5 d, SNRK-SMKO mice exhibited decreased MLCK expression in the colon without significant alterations in other contractile proteins. Similar expression profiles of these contractile proteins were also observed in the stomach and bladder compared to those in the colon. Myocardin is a master regulator of smooth muscle gene expression [48]. However, the deficiency of Snrk did not yield significant changes in myocardin protein expression observed in the colon or other detected tissues across mice aged from P3.5 d to P14.5 d. These results suggest that the increased expression levels of contractile proteins may represent a compensatory response to counteract the inadequate contractility resulting from loss of SMCs mediated by Snrk deficiency. Previous studies have demonstrated that Foxo4 can interact with myocardin, inhibiting its transcriptional activity [49]. Additionally, proteasome-mediated degradation of MLCK and SM22α has been implicated in contractile dysfunction and subsequent diseases [[50], [51], [52]]. Given the central role of the Myocardin-SRF axis in smooth muscle gene expression [39,40,48,53], we hypothesized that this pathway underlies the dynamics observed after SNRK loss. Time-series mRNA-protein correlation (P3.5 d-P14.5 d) and ChIP-qPCR for SRF/Myocardin occupancy at canonical promoters are needed to test this hypothesis. Despite the dynamic alteration observed in the expression of contractile proteins, the loss of Snrk inhibits the SMC expansion by consistently suppressing SMC proliferation. Further investigation is required to elucidate the underlying mechanism behind this inhibition. Moreover, broader evaluation of non-intestinal SMC beds is needed to determine whether extra-intestinal SMC defects are present and contribute to the premature death of SNRK-SMKO mice. Snrk has been identified to promote angioblast proliferation in zebrafish [15], while inhibiting colon cancer cell proliferation [23]. These findings suggest that the role of Snrk in regulating cell proliferation is context-dependent, varying according to the physiological or pathological conditions. Comprehensively assessing the movement and contractility of the intestinal tract at different development stages would provide valuable insights into the mechanisms by which Snrk regulates both intestinal development and function.

Currently, genetic studies focus on identifying new mutations within the CLMP and FLNA genes, initially discovered in patients with CSBS. Nevertheless, a substantial portion of patients have not undergone genetic examination, leaving the potential contribution of other gene mutations to CSBS largely unexplored. Human genetics analyses have revealed two single nucleotide polymorphisms within the SNRK gene associated with obesity risk in the United States population [19]. Mutations in SNRK have also been identified in affected tissues of patients with vascular anomalies [16]. In future, examining the presence of SNRK gene mutations will enhance our understanding of the genetic basis of CSBS.

## 5 Limitations of the study

Despite the new insights provided by our SMC-specific Snrk knockout models, several key questions remain. First, although we show that Snrk is essential for intestinal SMC proliferation, the specific downstream effectors and signaling pathways through which Snrk exerts this effect have not been defined and warrant further molecular investigation. Second, while we observe a clear proliferation defect in Snrk-deficient SMCs, the precise mechanism by which Snrk regulates cell-cycle progression remains to be elucidated. Finally, our findings are based exclusively on mouse models, so it is not yet known whether Snrk plays a similar role in human intestinal development or contributes to human CSBS. Clinical genetic screening of CSBS patients and functional studies in human tissues will be necessary to validate the translational relevance of our findings.

## Author contributions

M,H.Z. conceived the project. C.J.Y., M.H.Z., and Z.R.Z. designed the experiments. C.J.Y., L.O., J.A., and Y.D. performed the experiments and analyzed data. Z.X.L. provided critical technical assistance. C.J.Y. drafted the manuscript. M.H.Z. supervised the research and revised the manuscript. Z.R.Z. revised the manuscript. All authors have read and approved the final manuscript.

## Funding

This work was supported in part by funding from the 10.13039/501100001809National Natural Science Foundation of China (8247022344 to M.H.Z.) and the Transformational Award of American Heart Association (970764 to M.H.Z.).

## Declaration of competing interest

The authors declare no competing interests.

## Data Availability

Data will be made available on request.
