# Corrigendum to “Smooth muscle cell (SMC) Specific SNRK deletion in mouse causes congenital short bowel syndrome and premature death” [Biochem. Biophys. Rep. 44 (2025) 102298]

**DOI:** 10.1016/j.bbrep.2026.102484

**Published:** 2026-02-07

**Authors:** Chang-Jiang Yu, Liu Ouyang, Junqing An, Ye Ding, Zhi-Xue Liu, Zhi-Ren Zhang, Ming-Hui Zou

**Affiliations:** a Departments of Cardiology and Pharmacy, Harbin Medical University Cancer Hospital, Institute of Metabolic Disease, Heilongjiang Academy of Medical Science, Heilongjiang Key Laboratory for Metabolic Disorder and Cancer Related Cardiovascular Diseases, Harbin, China; b Center for Molecular and Translational Medicine, Georgia State University, Atlanta, USA; c Departments of Cardiology and Critical Care Medicine, The First Affiliated Hospital of Harbin Medical University, NHC Key Laboratory of Cell Transplantation, Key Laboratories of Education Ministry for Myocardial Ischemia Mechanism and Treatment, Harbin, China; d State Key Laboratory of Frigid Zone Cardiovascular Diseases (SKLFZCD), Harbin Medical University, Harbin, China; e Department of Endocrinology and Metabolism, Tianjin Medical University General Hospital, Tianjin, China

*Note to JM: Errors in article heads, particularly titles, author names, affiliations must not be repeated. In such cases, use the correct title, authors, affiliation(s), etc. If the corrigendum is the result of authorship changes, include the correct author details in the article head*.

The authors regret an error made in the funding information. Here is corrected information. This work was supported in part by funding from the National Natural Science Foundation of China (82470443 to M.H.Z.) and the Transformational Award of American Heart Association, USA (970764 to M.H.Z.). The authors would like to apologise for any inconvenience caused.

