# Supplementary material for: Smooth muscle cell (SMC)-Specific SNRK deletion in mouse causes congenital short bowel syndrome and premature death
Source: Biochem Biophys Rep. 2025 Oct 13;44:102298. doi: 10.1016/j.bbrep.2025.102298 (PMC12549929; doi:10.1016/j.bbrep.2025.102298)
Supplement: Multimedia component 1 [file mmc1.docx]

**Smooth Muscle Cell (SMC)-Specific SNRK Deletion in Mouse Causes Congenital Short Bowel Syndrome and Premature Death**

Chang-Jiang Yu ^a, b, 1^, Liu Ouyang ^b, 1^, Junqing An ^b^, Ye Ding ^b^, Zhi-Xue Liu ^b^, Zhi-Ren Zhang ^a, c, d, **^, Ming-Hui Zou ^b, e, *^

**Affiliation**

**a** Departments of Cardiology and Pharmacy, Harbin Medical University Cancer Hospital, Institute of Metabolic Disease, Heilongjiang Academy of Medical Science, Heilongjiang key laboratory for Metabolic disorder and cancer related cardiovascular diseases, Harbin, China

**b** Center for Molecular and Translational Medicine, Georgia State University, Atlanta, USA

**c** Departments of Cardiology and Critical Care Medicine, The First Affiliated Hospital of Harbin Medical University, NHC Key Laboratory of Cell Transplantation, Key Laboratories of Education Ministry for Myocardial Ischemia Mechanism and Treatment, Harbin, China

**d** State Key Laboratory of Frigid Zone Cardiovascular Diseases (SKLFZCD), Harbin Medical University, Harbin, China

**e** Department of Endocrinology and Metabolism, Tianjin Medical University General Hospital, Tianjin, China

***** Corresponding author. Department of Endocrinology and Metabolism, Tianjin Medical University General Hospital, 154 Anshan Road, Tianjin 300052, China.

****** Corresponding author. Departments of Cardiology and Pharmacy, Harbin Medical University Cancer Hospital, Institute of Metabolic Disease, Heilongjiang Academy of Medical Science, Heilongjiang key laboratory for Metabolic disorder and cancer related cardiovascular diseases, Harbin, China

E-mail addresses: mhzou@tmu.edu.cn (M.H.Z.), zhirenz@163.com (Z.R.Z.)

**1** These authors contributed equally


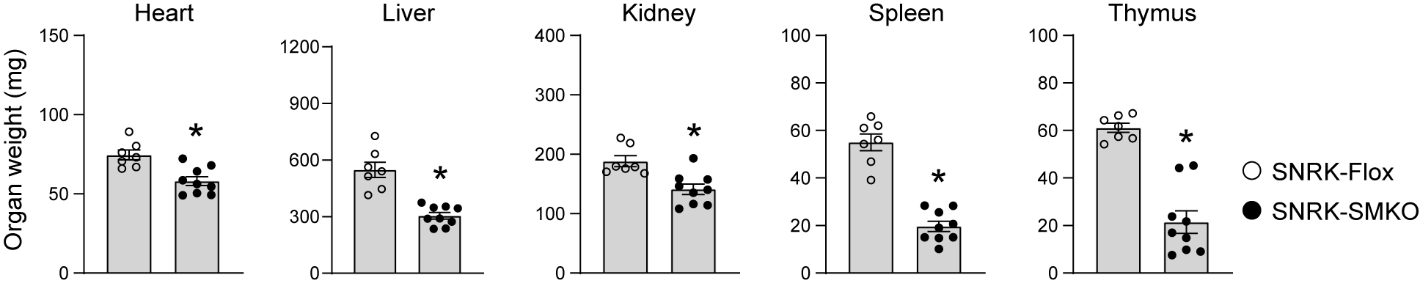


**Supplementary Fig. 1. Tissue weights in SNRK-Flox and SNRK-SMKO mice.** The weights of tissues, including heart, liver, kidney, spleen, and thymus were measured in SNRK-Flox and SNRK-SMKO mice under 25 d of age. n = 7 - 9 mice/group. **P* < 0.05 *vs*. SNRK-Flox group.


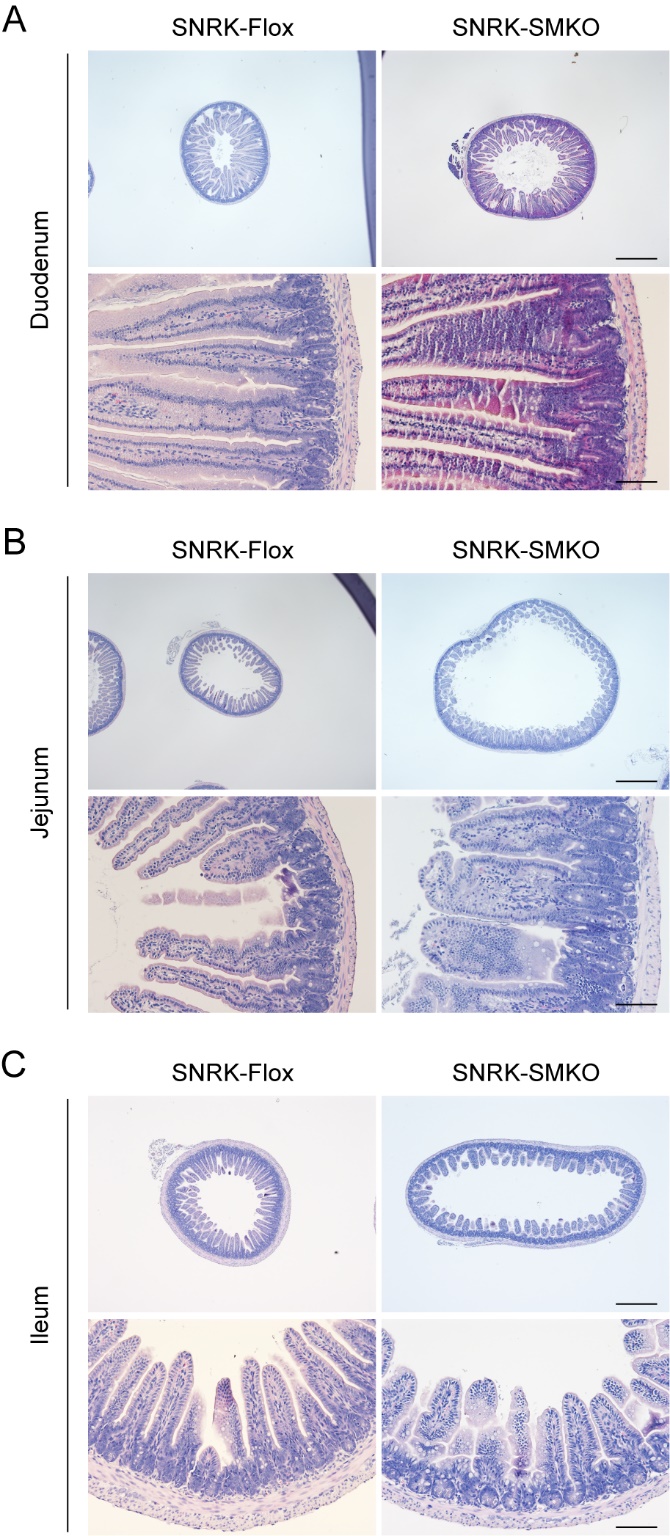


**Supplementary Fig. 2. Smooth muscle cell-specific Snrk gene deletion shows dilated small intestine and thinner circular smooth muscle layer.** Representative images of H&E staining of small intestine, including duodenum **(A)**, jejunum **(B)**, and ileum **(C)** cross sections from SNRK-Flox and SNRK-SMKO mice under 25 d of age. Scale bars, 1 mm for A and B (Top), 500 μm for C (Top). Scale bars, 100 μm for A - C (Bottom).


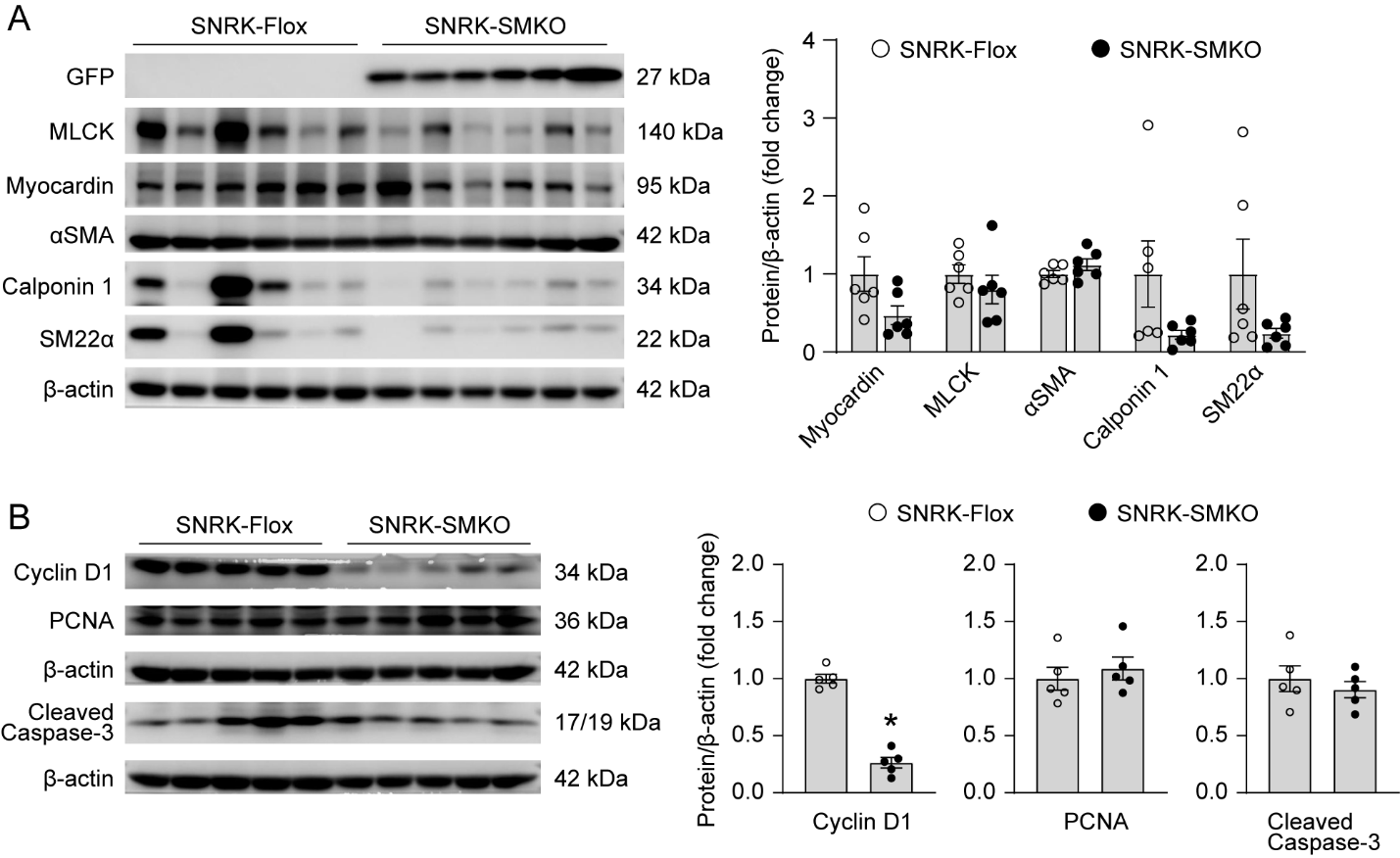


**Supplementary Fig. 3. Expression of contraction-, proliferation-, and apoptosis-related proteins in the bladder. (A)** Western blot analysis for the expression of contraction-related proteins in the bladders isolated from SNRK-Flox and SNRK-SMKO mice under 25 d of age. n = 6 mice/group. **(B)** Western blot analysis for the expression of Cyclin D1, PCNA, and Cleaved Caspase-3 in the bladders isolated from SNRK-Flox and SNRK-SMKO mice under 25 d of age. n = 5 mice/group. **P* < 0.05 *vs*. SNRK-Flox group.


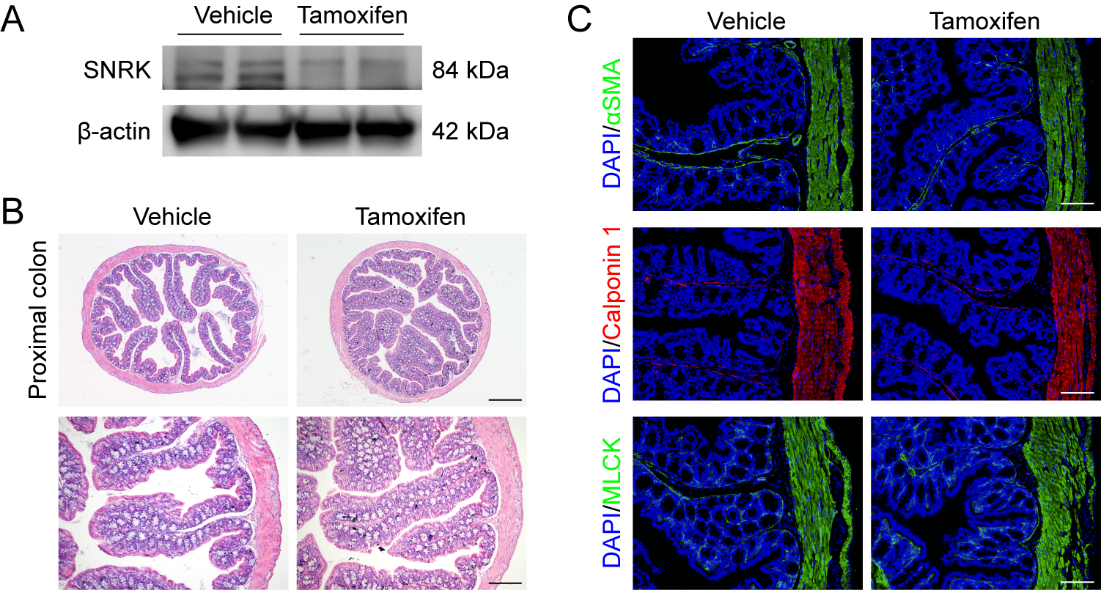


**Supplementary Fig. 4. Inducible smooth muscle cell-specific deletion of SNRK in adult mice results in normal intestinal morphology. (A)** Western blot result showed the reduction of Snrk expression in the colon smooth muscle of tamoxifen-treated Snrk- Myh11-CreER^T2^ mice. **(B)** Representative images of H&E staining of proximal colon. **(C)** Representative immunofluorescent images for αSMA, Calponin 1, and MLCK in the proximal colon.


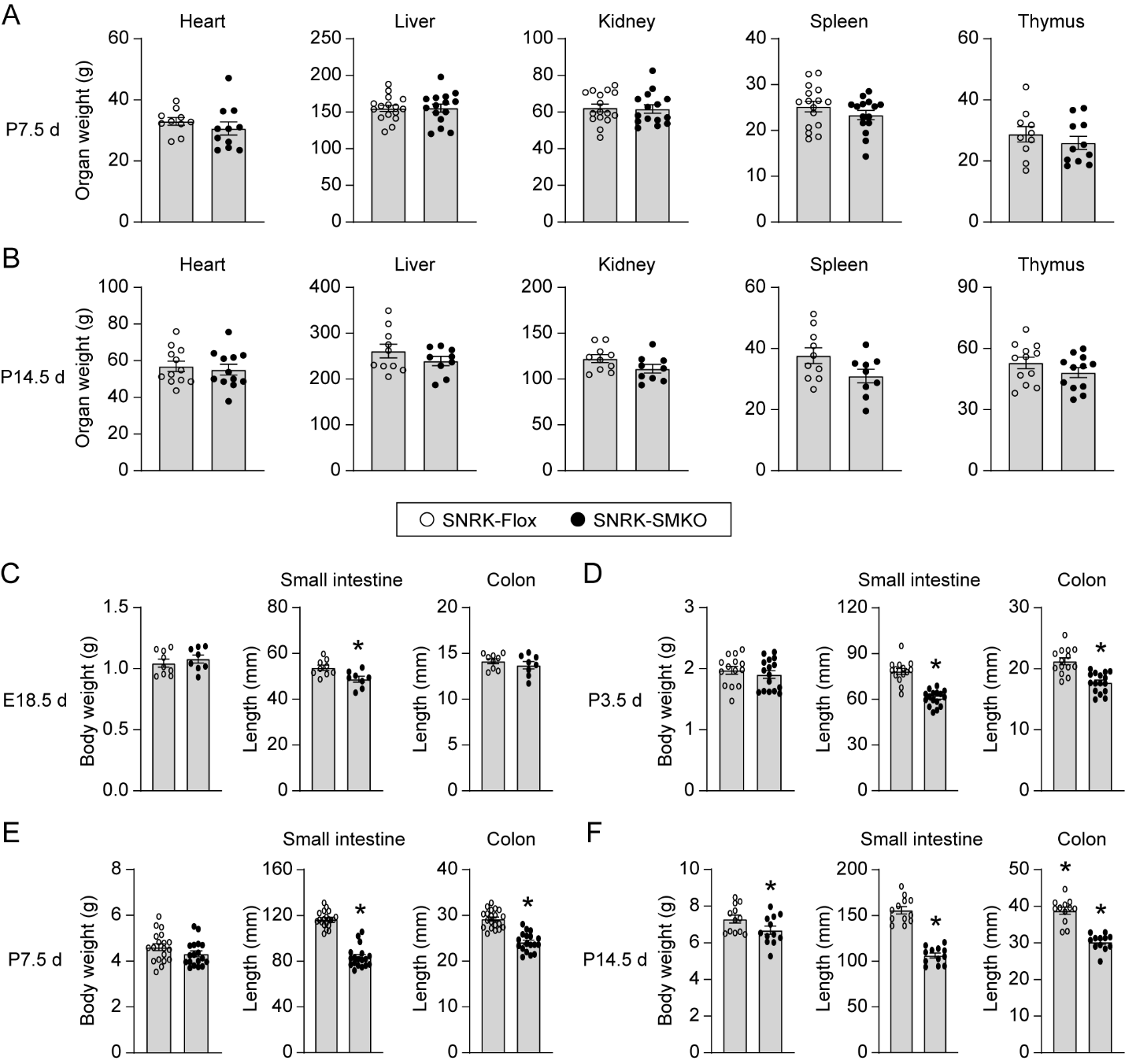


**Supplementary Fig. 5. Smooth muscle cell-specific SNRK gene deletion results in abnormal intestinal development. (A and B)** Tissue weights were measured in SNRK-Flox and SNRK-SMKO mice aged P7.5 d **(A)** and P14.5 d **(B)**. n = 10 - 16 mice/group in mice aged P7.5 d. n = 9 - 12 mice/group in mice aged P14.5 d. **(C - F)** Body weights, small intestine lengths, and colon lengths were measured in SNRK-Flox and SNRK-SMKO mice at the indicated ages. n = 8 - 9 mice/group in mice aged E18.5 d. n = 14 - 16 mice/group in mice aged P3.5 d. n = 18 - 20 mice/group in mice aged P7.5 d. n = 12 mice/group in mice aged P14.5 d.**P* < 0.05 *vs*. SNRK-Flox group.


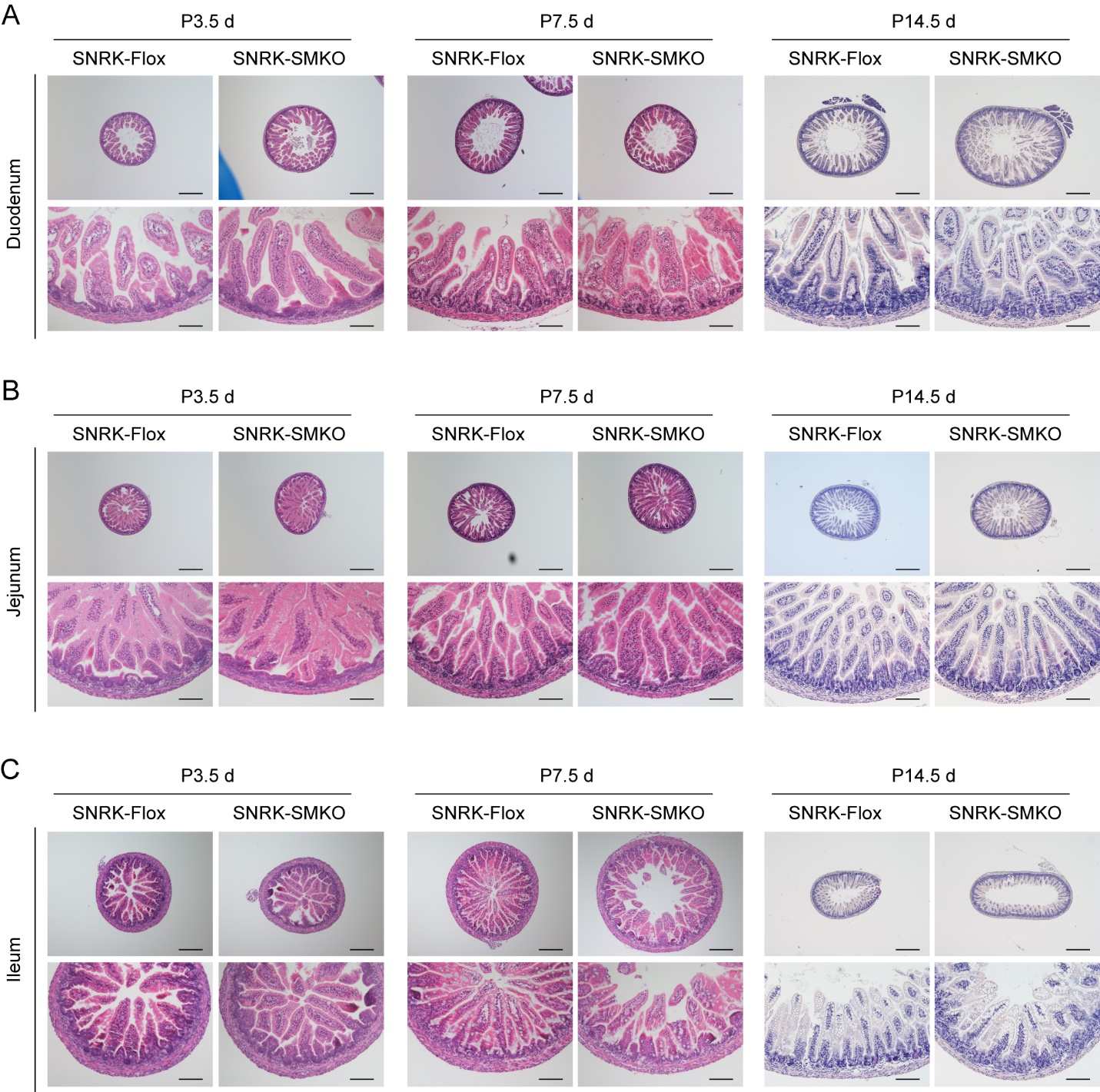


**Supplementary Fig. 6** **Smooth muscle cell-specific SNRK gene deletion decreases thickness of SMC layer in small intestine. (A - C)** Representative images of H&E staining of duodenum **(A)**, jejunum **(B)**, and ileum **(C)** in SNRK-Flox and SNRK-SMKO mice at the indicated ages.


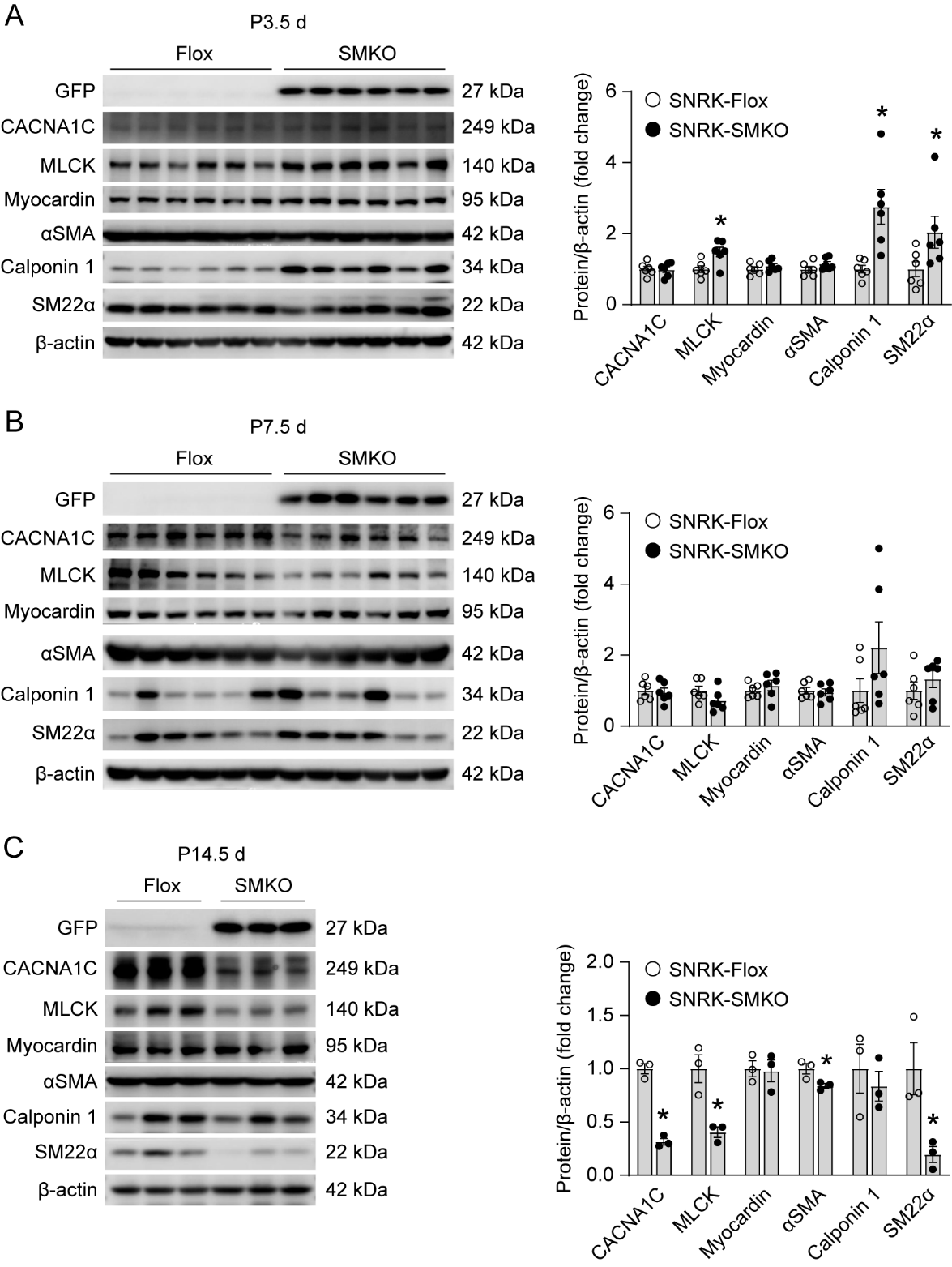


**Supplementary Fig. 7. The expression levels of smooth muscle contractile markers in the stomach.** Representative western blots and quantification of the expression levels of smooth muscle contractile makers in the stomach of SNRK-Flox and SNRK-SMKO mice at ages of P3.5 d **(A)**, P7.5 d **(B)**, and P14.5 d **(C)**. n = 6 mice/group in mice aged 3.5 d. n = 6 mice/group in mice aged P7.5 d. n = 3 mice/group in mice aged P14.5 d. **P* < 0.05 vs. SNRK-Flox group.


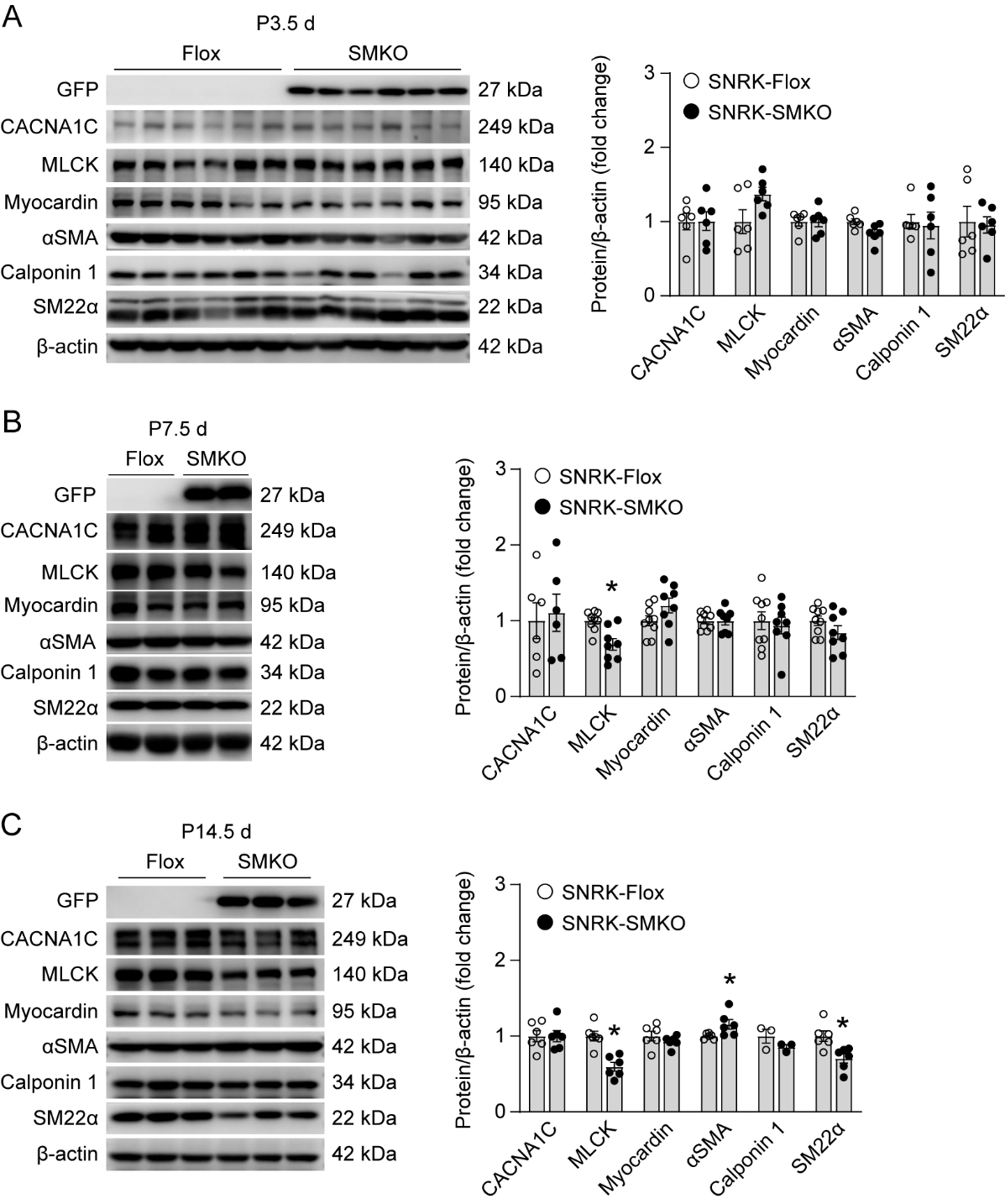


**Supplementary Fig. 8. The expression levels of smooth muscle contractile markers in the bladder.** Representative western blots and quantification of the expression levels of smooth muscle contractile makers in the bladder of SNRK-Flox and SNRK-SMKO mice at ages of P3.5 d **(A)**, P7.5 d **(B)**, and P14.5 d **(C)**. n = 6 mice/group in mice aged 3.5 d. n = 6 - 9 mice/group in mice aged P7.5 d. n = 3 - 6 mice/group in mice aged P14.5 d. **P* < 0.05 vs. SNRK-Flox group.


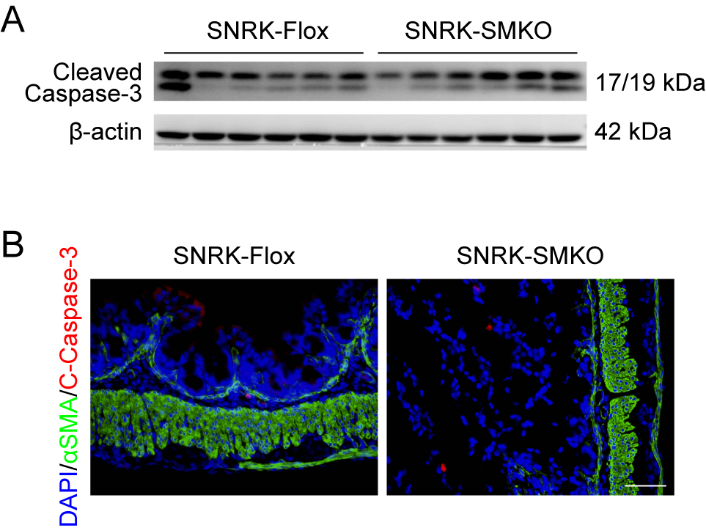


**Supplementary Fig. 9. Smooth muscle cell-specific SNRK gene deletion has no effect on SMC apoptosis. (A and B)** Representative western blot **(A)** and immunofluorescent image **(B)** showing cleaved caspase-3 expression in colon of SNRK-Flox and SNRK-SMKO mice at age of P7.5 d.
